# A new rat model of auxiliary partial heterotopic liver transplantation with liver dual arterial blood supply

**DOI:** 10.3892/etm.2014.2110

**Published:** 2014-12-05

**Authors:** JIANLIANG QIAO, CHUNLEI HAN, JUNJING ZHANG, ZHIYONG WANG, XINGKAI MENG

**Affiliations:** 1 Department of General Surgery, Xuanwu Hospital, Capital Medical University, Beijing 100053, P.R. China; 2 Department of General Surgery, The Affiliated Hospital of Inner Mongolia Medical University, Hohhot, Inner Mongolia Autonomous Region 010050, P.R. China; 3 Turku PET Centre, Turku University Hospital and University of Turku, Turku 20521, Finland

**Keywords:** auxiliary partial heterotopic liver transplantation, portal vein arterialization, liver dual arterial blood supply, rat

## Abstract

Auxiliary partial heterotopic liver transplantation (APHLT) with portal vein arterialization is a valuable procedure to be considered in the treatment of patients with acute liver failure and metabolic liver diseases. The aim of this study was to develop a new rat model of APHLT with liver dual arterial blood supply (LDABS). A total of 20 rats were used. The donor liver was resected, and the celiac trunk was reserved. Left and medial hepatic lobes accounting for 70% of the liver mass were removed en bloc and the suprahepatic caval vein was ligated simultaneously. Thus, 30% of the donor liver was obtained as the graft. Sleeve anastomosis of the graft portal vein and splenic artery were performed after narrowing the portal vein lumen through suturing. The right kidney of the recipient was removed, and sleeve anastomosis was performed between the celiac trunk of the graft and the right renal artery of the recipient. In addition, end-to-end anastomosis was performed between the infrahepatic caval vein of the graft and the right renal vein of the recipient. Following the reperfusion of the graft, the blood flow of the arterialized portal vein was controlled within the physiological range through suturing and narrowing under monitoring with an ultrasonic flowmeter. The bile duct of the graft was implanted into the duodenum of the recipient through an internal stent catheter. A 70% section of the native liver (left and medial hepatic lobes) was resected using bloodless hepatectomy. The mean operative duration was 154.5±16.4 min, and the warm and cold ischemia times of the graft were 8.1±1.1 min and 64.5±6.6 min, respectively. The blood flow of the arterialized portal vein to the graft was 1.8±0.3 ml/min/g liver weight. The success rate of model establishment (waking with post-surgical survival of >24 h) was 70% (7/10). Following successful model establishment, all rats survived 7 days post-surgery (100%; 7/7). The graft was found to be soft in texture and bright red in color following exploratory laparotomy. In conclusion, a new rat model of APHLT with LDABS without stent for vascular reconstruction was developed. This is a feasible and reliable rat model for liver transplantation study.

## Introduction

Auxiliary liver transplantation (ALT) is usually used for the treatment of acute liver failure and inherited metabolic liver disease (1,2). The aim of ALT is to support the patient’s failing liver for a period of time until the native liver has recovered, or to maintain normal metabolic functions using a small proportion of the liver mass. ALT has two main types, namely auxiliary partial orthotopic liver transplantation (APOLT) and auxiliary partial heterotopic liver transplantation (APHLT). APOLT is proposed to be a very effective option (1,2), whereas APHLT has been abandoned currently due to an increased incidence of primary non-function and portal vein thrombosis (3).

However, APHLT with portal vein arterialization (PVA) presents significant advantages, such as that the hilum and portal vein of the native liver are untouched, the surgical trauma of more extensive liver dissection of the native liver is avoided, and adequate portal venous blood flow of the graft liver is guaranteed. For these reasons, several clinical studies using APHLT with PVA have been conducted in recent years (4–6); however, the graft liver has been found to fail in long-term survival. To solve this problem, Schleimer *et al* developed a rat model of APHLT with PVA (7). In this model, the graft portal vein is connected by a stent with 0.3 mm inner diameter to the recipient’s right renal artery. The authors declared that the success rate was 90%; however, based on our experiences, thrombosis occurs quite frequently in the connected stent, despite the consistent use of systemic heparinization. This prompted the development of a new procedure to avoid the thrombosis. In the present study, it was hypothesized that the splenic artery in the celiac trunk from the graft liver could be used instead of a stent for PVA.

For this purpose, a new rat model of APHLT with PVA was developed, based on a modification of the Schleimer model, using the splenic artery of the graft liver for arterialization of the portal vein instead of a stent.

## Materials and methods

### Animals

The experimental protocol was approved by the Laboratory Animal Ethics Committee of the Inner Mongolia Medical University (Hohhot, China).

Male Sprague-Dawley (SD) rats, each weighing 300–350 g, were used in this study (obtained from Vital River Laboratory Animal Technology Co. Ltd, Beijing, China). The body weight of the donors and recipients were comparable. All procedures were performed by two persons using a clean but not sterile technique with a Leica M525 F20 surgical microscope (Leica Microsystems, Wetzlar, Germany). Anesthesia was induced by inhalation of 3–4% isoflurane, and maintained by inhalation of 1–2% isoflurane. All rats were given oxygen at a dose of 0.3–0.5 l/min.

### Donor surgery

#### Preparation of the donor liver

**Figure 1:**
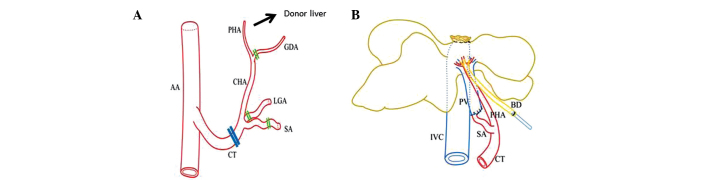
Schematic drawing of donor vessels and arterial blood supply to the graft liver. (A) Double lines indicate cutting positions. Note that SA is cut far from the CT. (B) Dual arterial blood supply of the graft liver. Note that the SA is connected to the PV. AA, abdominal aorta; PHA, proper hepatic artery; CHA, common hepatic artery; GDA, gastroduodenal artery; LGA, left gastric artery; SA, splenic artery; CT, celiac trunk; IVC, inferior vena cava; BD, bile duct; PV, portal vein.

After anesthesia was induced, a crucial incision was made in the abdomen of the rat. The ligament surrounding the liver was cut off with an electric coagulation scalpel, the left phrenic vein and right suprarenal vein were ligated and divided, and the infrahepatic caval vein was isolated and injected with 2 ml low molecular weight heparin sodium salt (50 IU/ml; Jiangsu Wanbang Biochemical Pharmaceutical, Co. Ltd, Xuzhou, China), allowing systemic heparinization of the rats. The hepatoduodenal ligament was dissected, and the common bile duct was isolated. The lower segment of the common bile duct was incised, and a stent catheter appropriately 1 cm in length (internal diameter, 0.5 mm) was implanted, followed by double ligature with 8-0 nylon sutures. The main portal vein was isolated, and the pyloric vein was ligated and divided. The celiac trunk and its branches were isolated, and the left gastric artery, splenic artery and gastroduodenal artery were ligated with 8-0 nylon sutures and divided. The splenic artery stump was retained at 5 mm in length at least for the subsequent sleeve anastomosis, and the common hepatic artery and proper hepatic artery were reserved (Fig. 1).

#### Perfusion of the donor liver

The donor liver was perfused through the portal vein. After clamping the origin of the celiac trunk, the distal portal vein was clamped and incised, and a perfusion catheter was implanted. Perfusion was performed with 30 ml lactated Ringer’s solution containing low molecular weight heparin sodium (12.5 IU/ml), using the gravity perfusion method at a rate of 45 drops/min at 0–4°C. The infrahepatic caval vein was rapidly incised to allow outflow of the blood and perfusate. The celiac trunk was transected at its origin and rinsed with lactated Ringer’s solution containing low molecular weight heparin sodium (12.5 IU/ml). During the perfusion, the donor liver was persistently rinsed with physiological saline at 0–4°C until the liver became khaki in color. The donor liver was completely resected and stored in lactated Ringer’s solution at 0–4°C.

#### Trimming of the donor liver

**Figure 2:**
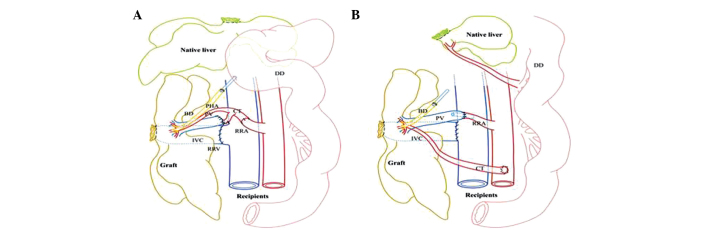
Comparison of vascular reconstruction in APHLT between this study and the Schleimer model. (A) Schematic drawing of LDABS in the current study. Note that the PV is connected to the SA directly and the CT is connected to the RRA without any stent. (B) Schematic drawing of the Schleimer model. Note that the PV is connected directly to the right renal artery using a stent, whereas the CT is connected to the AA by end-to-side anastomosis. APHLT, auxiliary partial heterotopic liver transplantation; LDABS, liver dual arterial blood supply; AA, abdominal aorta, PHA, proper hepatic artery; CHA, common hepatic artery; GDA, gastroduodenal artery; LGA, left gastric artery; SA, splenic artery; CT, celiac trunk; IVC, inferior vena cava; RRA, right renal artery; RRV, right renal vein; BD, bile duct; DD, duodenum; PV, portal vein.

The hepatic pedicle and hepatic vein of the left and medial liver lobes were ligated with 6-0 nylon sutures, and the left and medial hepatic lobes accounting for 70% of the liver mass were removed en bloc and the suprahepatic caval vein was ligated simultaneously. Thus, 30% of the donor liver was obtained as the graft. All lumens were rinsed with low molecular weight heparin sodium salt (50 IU/ml) and trimmed to facilitate the anastomosis. A portion of the lumen of the portal vein was sutured and closed with 10-0 nylon sutures to make the internal diameter of the portal vein lumen and external diameter of splenic artery comparable, and then sleeve anastomosis between the splenic artery and portal vein was performed (Fig. 2).

### Recipient surgery

#### Implantation of the donor liver

**Figure 3:**
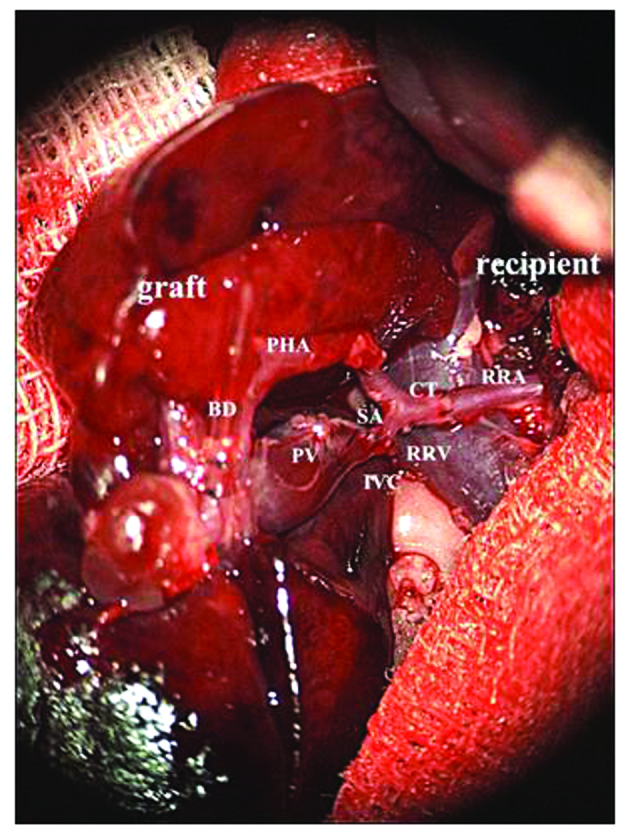
Microscopic aspect of vascular reconstruction of graft in auxiliary partial heterotopic liver transplantation. BD, bile duct; CT, celiac trunk; IVC, inferior vena cava; PHA, proper hepatic artery; PV, portal vein; RRA, right renal artery; RRV, right renal vein.

Following the successful induction of anesthesia, a median incision was made into the abdomen. The right renal artery and vein were isolated, and the origin of the right renal artery and the site for the right renal vein entry into the infrahepatic caval vein were clamped. The right kidney was then resected. The graft was implanted into the right renal fossa, and an inverted continuous all-layer suture was performed between the infrahepatic caval vein of the graft and the right renal vein of the recipient. Sleeve anastomosis was performed between the celiac trunk of the graft and the right renal artery of the recipient. Following successful anastomosis, the right renal venous clamp and right renal arterial clamp were removed successively to allow reperfusion; rapid recovery of the color of the graft, pulsation in the proper hepatic artery and celiac trunk, and cystic dilatation of the proximal portal vein were observed. The graft was warmed by rinsing with warm physiological saline, and bile was found to be discharged from the biliary stent catheter. Then, a stent catheter was implanted into the duodenum of the recipient, followed by purse-string suturing with 8-0 nylon sutures. To avoid dislocation and blood flow disturbance of the graft, the donor’s ligated suprahepatic caval vein was sutured and fixed with the right lateral abdominal wall (Fig. 3).

#### Blood flow control of arterialized transplanted hepatic portal vein

Following completion of the vascular reconstruction of the graft, the blood flow of the arterialized portal vein was measured using an ultrasonic flowmeter (Transonic Systems, Inc., Ithaca, NY, USA), and controlled within the physiological range (from 0.8±0.2 to 2.4±0.8 ml/min/g liver weight) (8) through suturing and narrowing the origin of the right renal artery of the recipient.

#### Recipient hepatectomy

A 70% section of the native liver (left and medial hepatic lobes) was resected en bloc by bloodless hepatectomy. The Glisson’s system of two hepatic lobes was ligated with 6-0 nylon sutures and then divided. The proximal portal vein was punctured and slowly infused with 3 ml lactated Ringer’s solution (37°C, similar to autotransfusion). Then, the hepatic vein was wrapped and ligated with 6-0 nylon sutures, and the left and medial hepatic lobes were resected successively in a clockwise direction. The abdominal cavity was infused with 5 ml warm lactated Ringer’s solution, and two-layer interrupted suture of the abdominal wall was performed with 3-0 silk sutures.

### Preoperative preparation

All rats were fasted for 24 h prior to the transplantation, and were given free access to water. During the transplantation, a heating blanket was applied to the recipient rats to keep the body temperature at 36°C. The recipient rats were awake within 3 min after the transplantation, and then were able to perform turn-over activities. A high post-operative body temperature was maintained, and all rats had free access to food and water. In addition, 2 ml low molecular weight heparin sodium salt (50 IU/ml) was injected subcutaneously daily following the surgery. Antibiotics and analgesics were not administered generally.

## Results

In total, 20 rats were used to establish the rat models of APHLT (10 as donors and 10 as recipients). The mean operative duration was 154.5±16.4 min, and the warm and cold ischemia times of the graft were 8.1±1.1 min and 64.5±6.6 min, respectively. The blood flow of the arterialized portal vein to the graft was 1.8±0.3 ml/min/g liver weight, as measured by the ultrasonic flowmeter, which was within the physiological range.

The blood flow at all anastomotic stomas was smooth following transplantation. Waking with a post-surgical survival of >24 h was defined as successful model establishment. In this study, the success rate of model establishment was 70% (7/10), and three rats died due to long operative duration, large trauma and bleeding of the anastomotic stoma in the 24 h after surgery. The rat had free access to food and water following the surgery. All rats were able to perform normal activities, had glossy hair, and were alert. All rats that were successfully modeled survived 7 days post-surgery (100%; 7/7). The graft was found to be soft in texture and bright red in color following exploratory laparotomy.

## Discussion

In the present study, a new rat model of APHLT with PVA was developed, characterized by: i) the graft splenic artery is reserved on the celiac trunk from the donor; ii) the graft portal vein is directly sleeve anastomosed to the graft splenic artery for arterialization of the portal vein; iii) the graft celiac trunk is sleeve anastomosed with the right kidney artery of the recipient; iv) the graft infrahepatic caval vein is end-to-end anastomosed with the right renal vein of the recipient. Furthermore, the study introduces a new term instead of PVA to describe this vascular reconstruction method; since the blood supplies of the graft liver in both the hepatic artery and portal vein are from same source (arterial blood from the graft celiac artery), the term is liver dual arterial blood supply (LDABS). LDABS differs from arterioportal fistula (9), partial portal vein arterialization (PPVA) (10) or arterialized portal vein blood supply alone during which the hepatic artery blood supply ceased owing to thrombosis, surgical ablation or injury (11,12).

Rat models have been widely used in experimental liver transplantation studies, including transplantation immunity, liver graft preservation and liver regeneration, since rats are cheap, and easy to model, as well as having immunological tolerance (13). The first ALT model in rats was reported by Lee and Edgington in 1966 (14). Subsequent to this, various ALT models in rats have been developed (15–17). Of these models, the APHLT with PVA model in rats was developed by Schleimer *et al* (7); one of advantages of this model is that portal venous blood flow is controlled by the connected stent. This model is simple and easy to perform. However, our experiments demonstrated that this method suffers from the following problems: i) the stent that joins the portal vein of the graft to the right renal artery of the recipient is extremely likely to undergo thrombosis, even if systemic heparinization is used following the surgery; ii) the abdominal aorta is required to be clamped during the end-to-side anastomosis between the celiac trunk of the graft and the recipient’s abdominal aorta for reconstruction of the hepatic artery in the graft liver. This procedure not only affects the recipient’s internal environment, but also is challenging to perform; iii) it is necessary to clamp the recipient’s infrahepatic caval vein during the end-to-side anastomosis with the infrahepatic caval vein of the graft, which further affects the rat’s systemic state.

To overcome these challenges, a new method for establishing the rat model of APHLT with LDABS was developed in the present study. This provided an experimental basis to investigate the feasibility of using LDABS to reconstruct the blood flow of the graft in heterotopic auxiliary liver transplantation and explore the molecular mechanism in the regulatory effect of LDABS on graft liver regeneration. The following aspects should be noted during the surgery: i) during the preparation of the donor liver, the isolation is difficult to perform due to the thin diameter of the celiac trunk and its branches, and the isolation may stimulate spasm of the proper hepatic artery, leading to the possible effect of transient ischemia on the donor liver; ii) the large differentiation in the diameter between the portal vein and splenic artery (~3-fold) leads to difficulties in anastomosis. In this study, a portion of the portal venous lumen was sutured and closed and then sleeve anastomosed with the splenic artery. The blood flow of the portal vein was 1.8±0.3 ml/min/g liver weight, as measured by the ultrasonic flowmeter, which was within the physiological range. However, the anastomosis is challenging to perform, and the operator should have a high-level microvascular anastomosis technique; iii) the hepatic artery and portal vein of the graft receive the same arterial blood supply from the recipient’ right renal artery via the celiac trunk of the graft, which avoids vascular reconstruction using the recipient’s abdominal aorta, and causes minor trauma to the recipient; and iv) end-to-end anastomosis of the infrahepatic caval vein of the graft and the recipient’s right renal vein is used to build the blood reflux pathway. This method may avoid the blockage of the recipient’s infrahepatic caval vein during the anastomosis, and reduce the effect on the recipient’s systemic state; however, this pathway is likely to develop venous return obstruction owing to vascular distortion. Therefore, the length of the infrahepatic caval vein of the graft and the recipient’s right renal vein should be shortened as much as possible so as to widen the venous outflow of the graft. Exploratory laparotomy one week after the surgery revealed that the graft was soft in texture and bright red in color.

In future, if the vascular reconstruction developed in this model is considered for application to large animals or even to human subjects, the iliac artery could be used instead of the renal artery, such as described by Fernández-Rodríguez *et al* in pigs (18).

This study has a limitation, which is that no histological analysis or serum tests of liver enzyme activity were performed to evaluate the functioning of the graft liver. Future studies to perform these are planned.

In conclusion, a new rat model of APHLT with LDABS without stenting for vascular reconstruction was developed. This is a feasible and reliable rat model for liver transplantation study.
